# Different packing motifs of isomeric (*E*)-*N*′-(halo­phenyl­methyl­idene)-*N*-methyl-2-(thio­phen-2-yl)acetohydrazides controlled by C—H⋯O inter­actions

**DOI:** 10.1107/S2056989018001251

**Published:** 2018-02-07

**Authors:** Laura N. F. Cardoso, Thais C. M. Noguiera, James L. Wardell, Marcus V. N. de Souza, William T. A. Harrison

**Affiliations:** a Fundação Oswaldo Cruz, Instituto de Tecnologia em Fármacos–FarManguinhos, Rua Sizenando Nabuco, 100, Manguinhos, 21041-250 Rio de Janeiro, Brazil; b Instituto de Química, Universidade Federal do Rio de Janeiro, Cidade Universitária, Rio de Janeiro, Brazil; c Department of Chemistry, University of Aberdeen, Meston Walk, Aberdeen AB24 3UE, Scotland

**Keywords:** crystal structure, carbohydrazide, methyl­ation, weak hydrogen bonds

## Abstract

The crystal structures of three isomeric (*E*)-*N*′-(chloro­phenyl­methyl­idene)-*N*-methyl-2-(thio­phen-2-yl)acetohydrazides (C_14_H_13_ClN_2_OS) are described, with the Cl atom in *ortho* (I), *meta* (III) and *para* (IV) positions in the benzene ring. The *ortho*-bromo derivative (II) (C_14_H_13_BrN_2_OS), which is isostructural with its chloro congener (I), is also reported. Mol­ecules (I)–(III) have similar conformations, which approximate to l-shapes, as indicated by their N—C—C—C_t_ (t = thio­phene) torsion angles of −90.1 (3), −91.44 (18) and −90.7 (9)°, respectively. The conformation of (IV) is different, with an equivalent torsion angle of −170.75 (11)° corresponding to a more extended shape for the mol­ecule. The thio­phene ring in each structure features ‘flip’ rotational disorder. The packing for (I) and (II) features inversion dimers, linked by pairs of C—H⋯O inter­actions, which generate *R*_2_^2^(14) loops. In the crystal of (III), [010] *C*(8) chains arise, with adjacent mol­ecules linked by pairs of C—H⋯O hydrogen bonds. The packing for (IV) features unusually short C—H⋯O inter­actions arising from an H atom attached to the benzene ring (H⋯O = 2.18 Å), which lead to *C*(9) [301] chains. Hirshfeld fingerprint percentage contact contributions are similar for the four title compounds.

## Abstract 2

The packing motifs in the isomeric title compounds feature inversion dimers or chains, mediated by C—H⋯O inter­actions, one of which is unusually short (H⋯O = 2.18 Å).

## Chemical context   

We have reported the syntheses and anti-TB activities of acetamido derivatives, 2-(*R*,*R*′NCOCH_2_)-thio­phene, *R* = alkyl (Nora de Souza *et al.*, 2008 ▸), and more recently thienyl aceto­hydrazide derivatives, 2-(ArCH=N—NHCOCH_2_)-thio­phene (Cardoso *et al.*, 2014 ▸). We are now studying the related family of methyl­ated 2-[ArCH=N—N(CH_3_)COCH_2_]-thio­phene compounds, with different substituents attached to the benzene ring. The biological activities of these compounds will be reported elsewhere: here, we present the crystal structures of three isomeric chloro derivatives (and one bromo derivative) in this family bearing a halogen atom at different sites on the benzene ring, *viz.* (*E*)-*N*′-(2-chloro­phenyl­methyl­idene)-*N*-methyl-2-(thio­phen-2-yl)acetohydrazide (I), (*E*)-*N*′-(2-bromo­phenyl­methyl­idene)-*N*-methyl-2-(thio­phen-2-yl)acetohydrazide (II), (*E*)-*N*′-(3-chloro­phenyl­methyl­idene)-*N*-methyl-2-(thio­phen-2-yl)acetohydrazide (III) and (*E*)-*N*′-(4-chloro­phenyl­methyl­idene)-*N*-methyl-2-(thio­phen-2-yl)acetohydrazide (IV). These complement our recent structural study (Cardoso *et al.*, 2016*a* ▸) of isomeric *ortho*-, *meta*- and *para*-nitro derivatives in the same family.
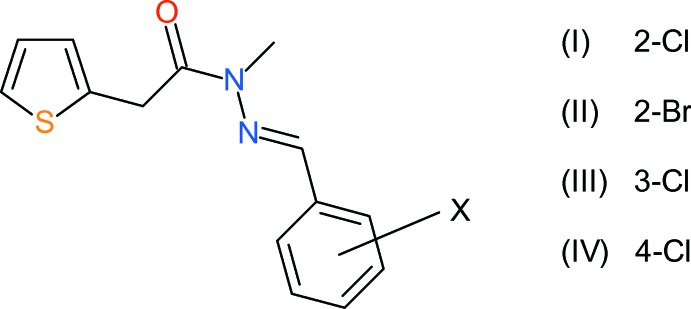


## Structural commentary   

**Figure 1:**
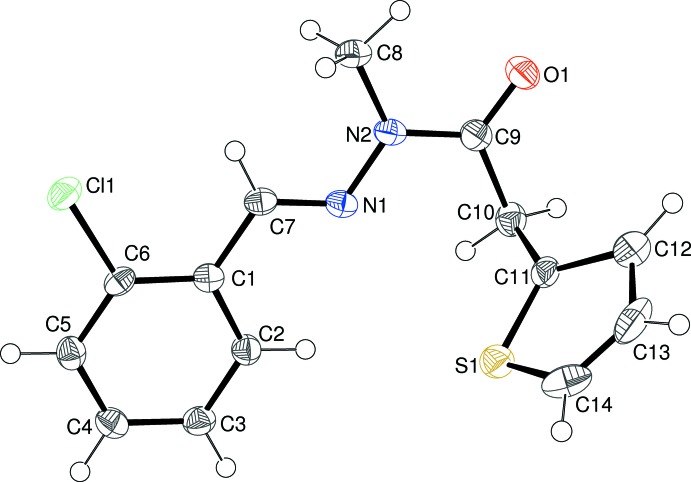
The mol­ecular structure of (I), showing 50% probability displacement ellipsoids. Only the major orientation of the thio­phene ring is shown.

The mol­ecular structure of (I) is shown in Fig. 1 ▸, which indicates that the expected methyl­ation has occurred at atom N2. The thio­phene ring (S1/C11–C14) shows ‘flip’ disorder (compare, for example, Sonar *et al.*, 2005 ▸; Wagner *et al.*, 2006 ▸) over two conformations rotated by ∼180° about the C10—C11 bond in a 0.658 (4):0.342 (4) ratio: the major orientation has the S atom pointing towards the C1–C6 benzene ring at the other end of the mol­ecule. The dihedral angle between the thio­phene and benzene rings is 77.92 (8)°. The central CH=N—N(CH_3_)—C(=O) fragment (C7/C8/C9/N1/N2/O1) in (I) is almost planar (r.m.s. deviation = 0.013 Å) and subtends dihedral angles of 0.89 (12) and 78.80 (9)° with the benzene and thio­phene rings, respectively. Thus, the major twist in the mol­ecule occurs about the C9—C10 bond [N2—C9—C10—C11 = −90.1 (3)°], giving the mol­ecule an approximate overall L-shape. The N1—N2 bond length of 1.372 (3)° is significantly shortened compared to the reference value of ∼1.41 Å for an isolated N—N single bond and the C9—N2 amide bond of 1.377 (3) Å is clearly lengthened: these data can be inter­preted in terms of significant delocalization of electrons over the methyl­idene–acetohydrazide group.

**Figure 2:**
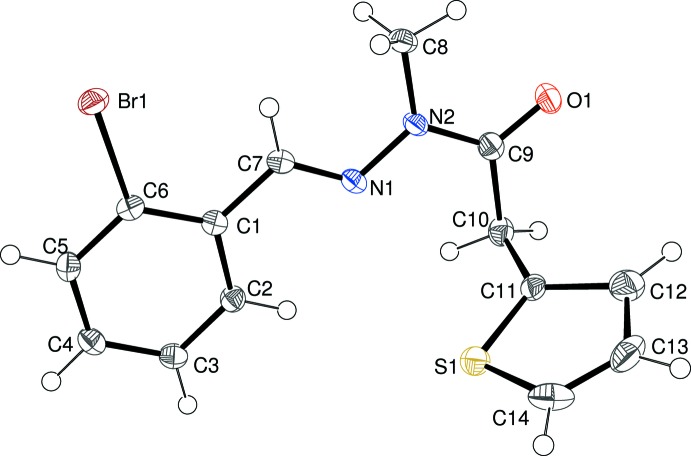
The mol­ecular structure of (II), showing 50% probability displacement ellipsoids. Only the major orientation of the thio­phene ring is shown.

Compound (II) (Fig. 2 ▸) is isostructural with (I); comparable geometrical data are as follows: C1–C6 benzene ring = ‘*A*’, thio­phene ring = ‘*B*’ [disorder occupancies = 0.677 (3):0.323 (3)], linking chain (r.m.s. deviation = 0.009 Å) = ‘*C*’; dihedral angles *A*/*B*, *A*/*C* and *B*/*C* = 75.89 (5), 1.53 (8) and 77.37 (6)°, respectively; N2—C9—C10—C11 = −91.44 (18)°, N1—N2 = 1.3720 (19) Å and C9—N2 = 1.375 (2) Å. These data are very similar to the corresponding values for (I); the only significant (and expected) difference is the C6—Br1 bond length of 1.9064 (16) Å in (II) compared to the C6—Cl1 distance of 1.748 (3) Å in (I).

**Figure 3:**
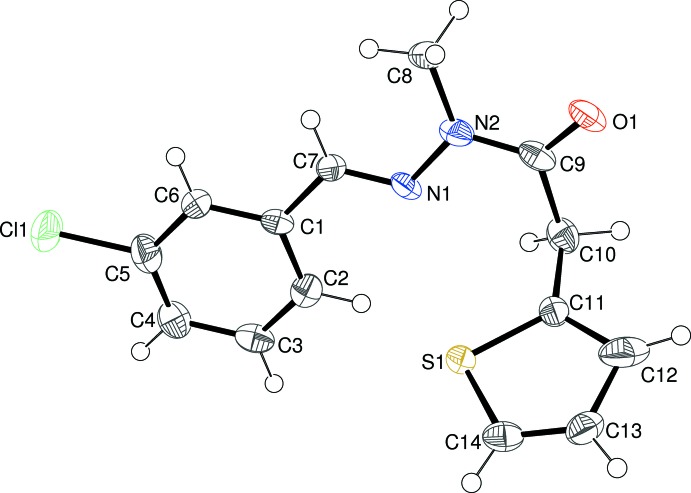
The mol­ecular structure of (III), showing 50% probability displacement ellipsoids. Only the major orientation of the thio­phene ring is shown.

The mol­ecular structure of (III) can be seen in Fig. 3 ▸: again the methyl­ation of N2 has occurred as expected. The dihedral angle between the thio­phene ring [rotationally disordered over two orientations in a 0.81 (1):0.19 (1) ratio] and the C1–C6 benzene ring is 66.0 (2)°. The approximately planar central C7/C8/C9/N1/N2/O1 group in (II) (r.m.s. deviation = 0.043 Å) subtends dihedral angles of 5.9 (5)° with the benzene ring and 62.9 (3)° with the thio­phene ring. As in (I) and (II), the major twist occurs about the C9—C10 bond [N2—C9—C10—C11 = −90.7 (9)°], giving the mol­ecule an approximate overall L-shape. The N1—N2 and C9—N2 bond lengths in (III) are 1.379 (9) and 1.363 (11) Å, respectively, which again can be ascribed to electronic effects.

**Figure 4:**
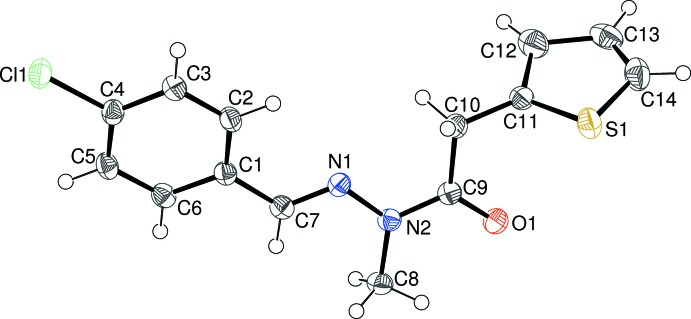
The mol­ecular structure of (IV), showing 50% probability displacement ellipsoids. Only the major orientation of the thio­phene ring is shown.

As with the other compounds, (IV) is methyl­ated at N2 (Fig. 4 ▸) and has a disordered thio­phene ring [major/minor disorder components = 0.671 (2):0.329 (2)]. The dihedral angles between the benzene ring ‘*A*’, thio­phene ring ‘*B*’ and CH=N—N(CH_3_)—C(=O)—CH_2_ fragment ‘*C*’ (r.m.s. deviation = 0.031 Å), are *A*/*B* = 81.82 (4), *A*/*C* = 14.79 (4) and *B*/*C* = 69.70 (5)°. These are roughly consistent with the equivalent data for (I)–(III), but the conformation of (IV) is definitely different, as indicated by the N2—C9—C10—C11 torsion angle of −170.75 (11)°: this reflects the fact that the thio­phene ring points away from the rest of the mol­ecule. Bond-length data [N1—N2 = 1.3778 (14) Å and C9—N2 = 1.3693 (16) Å] within the methyl­idene–acetohydrazide group for (IV) are consistent with the equivalent data for (I), (II) and (III).

## Supra­molecular features   

**Figure 5:**
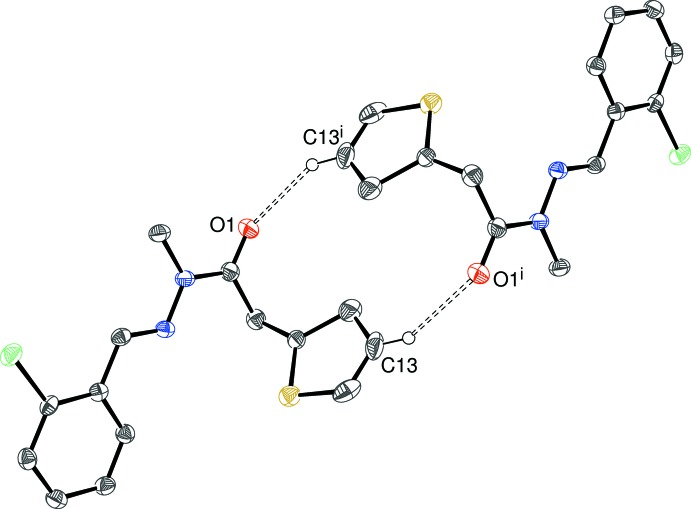
An inversion dimer in the crystal of (I) linked by a pair of C—H⋯O inter­actions. [Symmetry code: (i) −*x*, −*y*, 1 − *z*.] All H atoms except H13 have been omitted for clarity.

The packing motifs in (I) and (II) feature inversion dimers linked by pairs of C—H⋯O inter­actions (Fig. 5 ▸; Tables 1 ▸ and 2 ▸), with the C—H grouping part of the thio­phene ring: this generates an 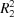
(14) loop. Weak C—H⋯π inter­actions consolidate the structures, but there are no aromatic π–π stacking inter­actions [minimum centroid–centroid separation = 4.86 Å for (I) and 4.85 Å for (II)].

**Figure 6:**
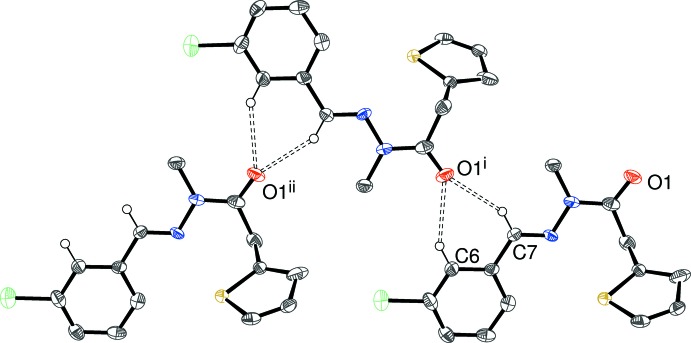
Fragment of an [010] hydrogen-bonded chain in the crystal of (III). [Symmetry codes: (i) 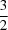 − *x*, *y* − 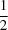, 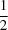 − *z*; (ii) *x*, *y* − 1, *z*.] All H atoms except H6 and H7 have been omitted for clarity.

The packing in (III) features two C—H⋯O inter­actions (Table 3 ▸) arising from benzene and adjacent methine C—H groups, which link the mol­ecules into [010] chains (Fig. 6 ▸), with adjacent mol­ecules in the chain related by the 2_1_ screw axis in the *b* direction. The C6 inter­action is long, but deemed to be just significant, as it is consolidating the C7 bond. Individually, each C—H⋯O bond generates a *C*(8) chain; collectively 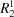
(6) loops arise. A very weak C—H⋯Cl bond is also observed. There are no C—H⋯π contacts in (III) and we consider that the shortest ring-centroid separation of 4.219 (5) Å is far too long to be regarded as a bonding inter­action.

**Figure 7:**
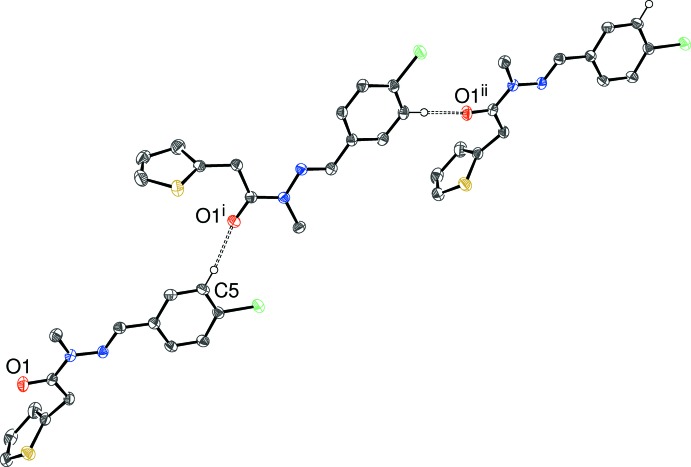
Fragment of a [301] hydrogen-bonded chain in the crystal of (IV). [Symmetry codes: (i) *x* − 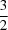, 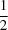 − *y*, *z* − 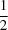; (ii) *x* − 3, *y*, *z* − 1.] All H atoms except H5 have been omitted for clarity.

In the crystal of (IV), an unusually short C—H⋯O inter­action (Table 4 ▸) with H⋯O = 2.18 Å leads to *C*(9) chains (Fig. 7 ▸) propagating in the [301] direction. The acceptor O atom deviates from the plane of Cl1/C4/C5/H5 by 0.239 (6) Å. One reason for the short contact could be the presence of the adjacent electron-withdrawing Cl substituent, which will tend to ‘activate’ the H atom (Steiner, 1996 ▸). Two extremely weak C—H⋯Cl inter­actions and a C—H⋯π contact occur, but there is no π–π stacking (minimum centroid–centroid separation = 4.42 Å) in the crystal of (IV).

Hirshfeld surface fingerprint plots for (I)–(IV) (supplementary Figs. 1 ▸–4 ▸ ▸ ▸) were calculated with *CrystalExplorer17* (Turner *et al.*, 2017 ▸) and percentage contact-surface contributions (McKinnon *et al.*, 2007 ▸) are listed in Table 5 ▸. As might be expected, the percentage contact data for the isomeric (I) and (II) are very similar but it is inter­esting that the data for (III) and (IV) barely differ from those of the first two compounds, despite their different crystal structures: in every case H⋯H contacts dominate the packing. This is quite different to the recently reported (*E*)-*N*′-(3-cyano­rophenyl­methyl­idene)-*N*-methyl-2-(thio­phen-2-yl)acetohydrazide (V) and (*E*)-*N*′-(4-meth­oxy­phenyl­methyl­idene)-*N*-methyl-2-(thio­phen-2-yl)acetohydrazide (VI) (Cardoso *et al.*, 2017 ▸), where the percentage contributions of the different inter­molecular contacts to the fingerprint plots differ by up to 20%.

## Database survey   

A survey of the Cambridge Structural Database (Groom *et al.*, 2016 ▸) updated to June 2017 for the common central —CH=N—N(CH_3_)—C(=O)—CH_2_— fragment of the title compounds revealed seven matches, *viz.* ALAHEC (Cardoso *et al.*, 2016*b* ▸); FOTMUX (Ramírez *et al.*, 2009*a* ▸); KULREP (Ramírez *et al.*, 2009*b* ▸); OFEBIL (Cao *et al.*, 2007 ▸), and EYUBAD, EYUBEH and EYUBIL; this latter trio of refcodes correspond to the three isomeric nitro compounds (Cardoso *et al.*, 2016*a* ▸) noted in the *Chemical Context* section above. To this list will soon be added the structures of (V) and (VI) noted above.

## Synthesis and crystallization   

The appropriate thienyl acetohydrazide derivative (Cardoso *et al.*, 2014 ▸) (0.20 g, 1.0 equiv.) was suspended in acetone (5 ml) and potassium carbonate (4.0 equiv.) was added. The reaction mixture was stirred at room temperature for 30 min. and methyl iodide (4.0 equiv.) was added. The reaction mixture was maintained at 313 K, until thin-layer chromatography indicated the reaction was complete. The mixture was then rotary evaporated to leave a residue, which was dissolved in water (20 ml) and extracted with ethyl acetate (3 × 10 ml). The organic fractions were combined, dried with anhydrous MgSO_4_, filtered and the solvent evaporated at reduced pressure. The crystals used for the intensity data collections were recrystallized from methanol solution.

(*E*)-*N*′-(2-Chloro­phenyl­methyl­idene)-*N*-methyl-2-(thio­phen-2-yl)acetohydrazide (I); yield: 66%; yellow solid; m.p. 91–92 °C. ^1^H NMR (400 MHz; DMSO): δ 8.10–8.08 (2H; *m*; H-11′ and N=CH), 7.57–7.54 (1H; *m*; H-8′), 7.47–7.44 (2H; *m*; H-9′ and H-10′), 7.36 (1H; *dd*; *J*_HH_ = 4.1 and 1.0 Hz; H-5), 6.98–6.97 (1H; *m*; H-4), 6.96–6.94 (1H; *m*; H-3), 4.39 (2H; *s*; CH_2_), 3.36 (3H; *s*; N-CH_3_). ^13^C NMR (125 MHz; DMSO): δ 171.0 (C=O), 136.9 (N=CH), 135.8 (C-2), 133.2 (C-6′), 131.6 (C-7′), 131.1 (C-9′), 129.9 (C-8′), 127.6 (C-11′), 127.2 (C-10′), 126.7 (C-3), 126.5 (C-4), 125.2 (C-5), 34.2 (N–CH_3_), 28.0 (CH_2_). MS/ESI: [*M* + Na]: 315. IR ν_max_ (cm^−1^; KBr pellet): 1680 (C=O); 3689 (N–CH_3_).

(*E*)-*N*′-(2-Bromo­phenyl­methyl­idene)-*N*-methyl-2-(thio­phen-2-yl)acetohydrazide (II); yield: 70%; yellow solid; m.p. 87–88 °C. ^1^H NMR (400 MHz; DMSO): δ 8.07 (1H; *dd*; *J*_HH_ = 7.6 and 1.6 Hz; H-11′), 8.03 (1H; *s*; N=CH), 7.72 (1H; *dd*; *J*_HH_ = 8.0 and 0.8 Hz; H-8′), 7.49 (1H; *t*; *J*_HH_ = 7.6 Hz; H-10′), 7.39–7.35 (2H; *m*; H-9′ and H-5), 6.98–6.94 (2H; *m*; H-3 and H-4), 4.39 (2H; *s*; CH_2_), 3.36 (3H; *s*; N-CH_3_). ^13^C NMR (125 MHz; DMSO): δ 171.0 (C=O), 138.1 (N=CH), 136.9 (C-2), 133.2 (C-6′), 133.0 (C-8′), 131.4 (C-9′), 128.1 (C-3), 127.6 (C-10′ and C-11′), 126.7 (C-4), 125.2 (C-5), 123.5 (C-7′), 34.2 (N-CH_3_), 28.0 (CH_2_). MS/ESI: [*M* + Na]: 359. IR ν_max_ (cm^−1^; KBr pellet): 1680 (C=O); 3676 (N–CH_3_).

(*E*)-*N*′-(3-Chloro­phenyl­methyl­idene)-*N*-methyl-2-(thio­phen-2-yl)acetohydrazide (III): yield: 64%; yellow solid; m.p. 120–121 °C. ^1^H NMR (500 MHz, DMSO): δ 7.97 (1H; *s*; CH=N), 7.80 (2H; *d*; *J*_HH_ = 9.0 Hz; C_6_H_6_), 7.50 (2H; *d*; *J*_HH_ = 8.5Hz; C_6_H_6_), 7.30 (1H; *dd*; *J*_HH_ = 5.5 and 1.5 Hz; H-5), 6.97 (1H; *d*; *J*_HH_ = 2.5Hz; H-3), 6.94–6.93 (1H; *m*; H-4), 4.37 (2H; *s*; CH_2_), 3.34 (3H; *s*; CH_3_). ^13^C NMR (500 MHz, DMSO): δ 170.3 (C-2′), 138.8 (CH=N), 136.7 (C-2), 133.7 (phen­yl), 133.3 (phen­yl), 128.3 (phen­yl), 128.2 (C_6_H_6_), 126.0 (C-4), 125.9 (C-3), 124.3 (C-5), 33.8 (CH_3_), 27.6 (CH_2_). IR ν_max_ (cm^−1^; KBr pellet): 1681 (C=O); 3715 (N–CH_3_).

(*E*)-*N*′-(4-Chloro­phenyl­methyl­idene)-*N*-methyl-2-(thio­phen-2-yl)acetohydrazide (IV); yield: 55%; yellow solid; m.p. 121–122 °C. ^1^H NMR (400 MHz; DMSO): δ 8.00 (1H; *s*; N=CH), 7.84 (2H; *d*; *J*_HH_ = 8.4 Hz; H-7′ and H-11′), 7.54 (2H; *d*; *J*_HH_ = 8.4 Hz; H-8′ and H-10′), 7.35 (1H; *dd*; *J*_HH_ = 4.8 and 0.8 Hz; H-5), 6.98–6.93 (2H; *m*; H-3 and H-4), 4.36 (2H; *s*; CH_2_), 3.32 (3H; *s*; N-CH_3_). ^13^C NMR (125 MHz; DMSO): δ 170.7 (C=O), 139.4 (N=CH), 137.0 (C-2), 134.1 (C-9′), 133.6 (C-6′), 128.8 (C-7′ and C-11′), 128.7 (C-8′ and C-10′), 126.6 (C-3), 126.5 (C-4), 125.1 (C-5), 34.2 (N-CH_3_), 28.0 (CH_2_). MS/ESI: [*M* + Na]: 315. IR ν_max_ (cm^−1^; KBr pellet): 1680 (C=O); 3689 (N–CH_3_).

## Refinement   

Crystal data, data collection and structure refinement details are summarized in Table 6 ▸. H atoms were placed geometrically (C—H = 0.95–1.00 Å) and refined as riding atoms. The constraint *U*_iso_(H) = 1.2*U*_eq_(carrier) or 1.5*U*_eq_(meth­yl) was applied in all cases. The *N*-methyl group was allowed to rotate, but not to tip, to best fit the electron density (AFIX 137 instruction in *SHELXL*; Sheldrick, 2015 ▸); in every case, this group rotated from its intial calculated orientation to minimize steric inter­action with H7; the final optimized geometry leads to a short (H⋯O ∼ 2.35 Å) intra­molecular C8—H⋯O1 contact but we do not regard this as a bond. The thio­phene rings show ∼180° ‘flip’ rotational disorder about the C10—C11 bond for all compounds. The crystal of (III) used for data collection was small and data quality was poor. Iin the refinement, difference maps indicated significant unmodelled electron density in the vicinity of C4. This was modelled in terms of a minor impurity/disorder component with the Cl atom bonded to C4 rather than C3. Even after the disorder modelling, the residuals are high, but we deem the refinement to be acceptable in terms of its chemical information content.

## Supplementary Material

Crystal structure: contains datablock(s) I, II, III, IV, global. DOI: 10.1107/S2056989018001251/sj5543sup1.cif

Structure factors: contains datablock(s) I. DOI: 10.1107/S2056989018001251/sj5543Isup2.hkl

Structure factors: contains datablock(s) II. DOI: 10.1107/S2056989018001251/sj5543IIsup3.hkl

Structure factors: contains datablock(s) III. DOI: 10.1107/S2056989018001251/sj5543IIIsup4.hkl

Structure factors: contains datablock(s) IV. DOI: 10.1107/S2056989018001251/sj5543IVsup5.hkl

CCDC references: 1818231, 1818230, 1818229, 1818228

Additional supporting information:  crystallographic information; 3D view; checkCIF report

## Figures and Tables

**Table 1:** Hydrogen-bond geometry (Å, °) for (I) *Cg*1 is the centroid of the thio­phene ring, *Cg*2 is the centroid of the benzene ring.

*D*—H⋯*A*	*D*—H	H⋯*A*	*D*⋯*A*	*D*—H⋯*A*
C13—H13⋯O1^i^	0.95	2.54	3.410 (4)	153
C3—H3⋯*Cg*1^ii^	0.95	2.83	3.612 (3)	140
C8—H8*A*⋯*Cg*2^iii^	0.98	2.71	3.544 (3)	144

Symmetry codes: (i) 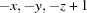; (ii) 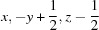; (iii) 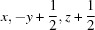.

**Table 2:** Hydrogen-bond geometry (Å, °) for (II) *Cg*1 is the centroid of the thio­phene ring, *Cg*2 is the centroid of the benzene ring.

*D*—H⋯*A*	*D*—H	H⋯*A*	*D*⋯*A*	*D*—H⋯*A*
C13—H13⋯O1^i^	0.95	2.53	3.424 (2)	156
C3—H3⋯*Cg*1^ii^	0.95	2.82	3.6021 (18)	141
C8—H8*A*⋯*Cg*2^iii^	0.98	2.67	3.495 (2)	142

Symmetry codes: (i) 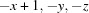; (ii) 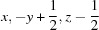; (iii) 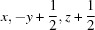.

**Table 3:** Hydrogen-bond geometry (Å, °) for (III)

*D*—H⋯*A*	*D*—H	H⋯*A*	*D*⋯*A*	*D*—H⋯*A*
C6—H6⋯O1^i^	0.95	2.62	3.474 (9)	150
C7—H7⋯O1^i^	0.95	2.52	3.381 (9)	152
C12—H12⋯Cl1^ii^	0.95	2.83	3.415 (7)	121

Symmetry codes: (i) 
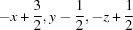
; (ii) 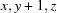.

**Table 4:** Hydrogen-bond geometry (Å, °) for (IV) *Cg*1 is the centroid of the benzene ring.

*D*—H⋯*A*	*D*—H	H⋯*A*	*D*⋯*A*	*D*—H⋯*A*
C5—H5⋯O1^i^	0.95	2.18	3.1250 (15)	172
C3—H3⋯Cl1^ii^	0.95	2.95	3.8044 (14)	151
C12—H12⋯Cl1^iii^	0.95	2.98	3.7960 (10)	145
C8—H8*C*⋯*Cg*1^iv^	0.98	2.73	3.5592 (14)	142

Symmetry codes: (i) 
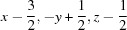
; (ii) 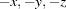; (iii) 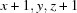; (iv) 
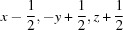
.

**Table 5:** Hirshfeld contact inter­actions (%)

Contact type	(I)	(II)	(III)	(IV)
H⋯H	43.6	43.0	38.5	41.5
C⋯H/H⋯C	21.3	20.8	18.1	23.5
Hal⋯H/H⋯Hal	12.5	13.0	15.2	16.0
O⋯H/H⋯O	9.4	9.6	9.7	7.1
C⋯C	2.5	2.4	4.7	1.6
N⋯H/H⋯N	1.4	1.3	3.9	3.3
S⋯H/H⋯S	1.9	1.8	2.9	2.0

**Table 6:** Experimental details

	(I)	(II)	(III)	(IV)
Crystal data				
Chemical formula	C_14_H_13_ClN_2_OS	C_14_H_13_BrN_2_OS	C_14_H_13_ClN_2_OS	C_14_H_13_ClN_2_OS
*M*_r_	292.77	337.23	292.77	292.77
Crystal system, space group	Monoclinic, *P*2_1_/*c*	Monoclinic, *P*2_1_/*c*	Monoclinic, *P*2_1_/*n*	Monoclinic, *P*2_1_/*n*
Temperature (K)	100	100	100	100
*a*, *b*, *c* (Å)	9.1918 (7), 20.3575 (14), 7.2721 (5)	9.4479 (7), 20.2175 (14), 7.2552 (5)	4.2194 (2), 13.0131 (9), 25.0758 (18)	6.7454 (5), 20.2993 (14), 10.1592 (7)
β (°)	96.360 (2)	96.9343 (13)	93.752 (4)	97.510 (2)
*V* (Å^3^)	1352.40 (17)	1375.70 (17)	1373.90 (15)	1379.14 (17)
*Z*	4	4	4	4
Radiation type	Mo *K*α	Mo *K*α	Mo *K*α	Mo *K*α
μ (mm^−1^)	0.43	3.13	0.42	0.42
Crystal size (mm)	0.53 × 0.24 × 0.18	0.26 × 0.06 × 0.05	0.22 × 0.01 × 0.01	0.30 × 0.17 × 0.10

Data collection				
Diffractometer	Rigaku Mercury CCD	Rigaku Mercury CCD	Rigaku Mercury CCD	Rigaku Mercury CCD
Absorption correction	Multi-scan (*SADABS*; Bruker, 2004 ▸)	Multi-scan (*SADABS*; Bruker, 2004 ▸)	Multi-scan (*SADABS*; Bruker, 2004 ▸)	Multi-scan (*SADABS*; Bruker, 2004 ▸)
*T*_min_, *T*_max_	0.710, 1.000	0.756, 1.000	0.615, 1.000	0.843, 1.000
No. of measured, independent and observed [*I* > 2σ(*I*)] reflections	18992, 2988, 2553	14778, 3163, 2897	12868, 3121, 1766	14763, 3165, 2929
*R*_int_	0.032	0.040	0.127	0.033
(sin θ/λ)_max_ (Å^−1^)	0.649	0.650	0.648	0.650

Refinement				
*R*[*F* ^2^ > 2σ(*F* ^2^)], *wR*(*F* ^2^), *S*	0.052, 0.117, 1.22	0.025, 0.064, 1.06	0.116, 0.297, 1.16	0.033, 0.091, 1.05
No. of reflections	2988	3163	3121	3165
No. of parameters	175	174	184	174
H-atom treatment	H-atom parameters constrained	H-atom parameters constrained	H-atom parameters constrained	H-atom parameters constrained
Δρ_max_, Δρ_min_ (e Å^−3^)	0.37, −0.41	0.48, −0.43	1.00, −0.45	0.53, −0.40

Computer programs: *CrystalClear* (Rigaku, 2012 ▸), *SHELXS97* (Sheldrick, 2008 ▸), *SHELXL2014* (Sheldrick, 2015 ▸), *ORTEP-3* (Farrugia, 1997 ▸) and *publCIF* (Westrip, 2010 ▸).

## supplementary crystallographic information

### (E)-N'-(2-Chlorophenylmethylidene)-N-methyl-2-(thiophen-2-yl)acetohydrazide (I) Crystal data

**Table:**

C_14_H_13_ClN_2_OS	*F*(000) = 608
*M**_r_* = 292.77	*D*_x_ = 1.438 Mg m^−^^3^
Monoclinic, *P*2_1_/*c*	Mo *K*α radiation, λ = 0.71073 Å
*a* = 9.1918 (7) Å	Cell parameters from 18890 reflections
*b* = 20.3575 (14) Å	θ = 2.0–27.5°
*c* = 7.2721 (5) Å	µ = 0.43 mm^−^^1^
β = 96.360 (2)°	*T* = 100 K
*V* = 1352.40 (17) Å^3^	Block, colourless
*Z* = 4	0.53 × 0.24 × 0.18 mm

### (E)-N'-(2-Chlorophenylmethylidene)-N-methyl-2-(thiophen-2-yl)acetohydrazide (I) Data collection

**Table:**

Rigaku Mercury CCD diffractometer	2553 reflections with *I* > 2σ(*I*)
ω scans	*R*_int_ = 0.032
Absorption correction: multi-scan (SADABS; Bruker, 2004)	θ_max_ = 27.5°, θ_min_ = 2.2°
*T*_min_ = 0.710, *T*_max_ = 1.000	*h* = −11→11
18992 measured reflections	*k* = −26→26
2988 independent reflections	*l* = −9→9

### (E)-N'-(2-Chlorophenylmethylidene)-N-methyl-2-(thiophen-2-yl)acetohydrazide (I) Refinement

**Table:**

Refinement on *F*^2^	Hydrogen site location: inferred from neighbouring sites
Least-squares matrix: full	H-atom parameters constrained
*R*[*F*^2^ > 2σ(*F*^2^)] = 0.052	*w* = 1/[σ^2^(*F*_o_^2^) + (0.0254*P*)^2^ + 1.9705*P*] where *P* = (*F*_o_^2^ + 2*F*_c_^2^)/3
*wR*(*F*^2^) = 0.117	(Δ/σ)_max_ < 0.001
*S* = 1.22	Δρ_max_ = 0.37 e Å^−^^3^
2988 reflections	Δρ_min_ = −0.41 e Å^−^^3^
175 parameters	Extinction correction: SHELXL-2014/7 (Sheldrick 2015), Fc^*^=kFc[1+0.001xFc^2^λ^3^/sin(2θ)]^-1/4^
0 restraints	Extinction coefficient: 0.0035 (9)
Primary atom site location: structure-invariant direct methods	

### (E)-N'-(2-Chlorophenylmethylidene)-N-methyl-2-(thiophen-2-yl)acetohydrazide (I) Special details

**Table:**

Geometry. All esds (except the esd in the dihedral angle between two l.s. planes) are estimated using the full covariance matrix. The cell esds are taken into account individually in the estimation of esds in distances, angles and torsion angles; correlations between esds in cell parameters are only used when they are defined by crystal symmetry. An approximate (isotropic) treatment of cell esds is used for estimating esds involving l.s. planes.

### (E)-N'-(2-Chlorophenylmethylidene)-N-methyl-2-(thiophen-2-yl)acetohydrazide (I) Fractional atomic coordinates and isotropic or equivalent isotropic displacement parameters (Å^2^)

**Table:**

	*x*	*y*	*z*	*U*_iso_*/*U*_eq_	Occ. (<1)
C1	0.4009 (3)	0.30066 (11)	0.2999 (3)	0.0205 (5)	
C2	0.2682 (3)	0.32749 (12)	0.2216 (4)	0.0238 (5)	
H2	0.1913	0.2989	0.1741	0.029*	
C3	0.2462 (3)	0.39464 (12)	0.2116 (4)	0.0261 (5)	
H3	0.1551	0.4117	0.1578	0.031*	
C4	0.3576 (3)	0.43717 (12)	0.2804 (4)	0.0264 (6)	
H4	0.3421	0.4833	0.2749	0.032*	
C5	0.4909 (3)	0.41239 (12)	0.3568 (3)	0.0249 (5)	
H5	0.5680	0.4413	0.4017	0.030*	
C6	0.5107 (3)	0.34485 (12)	0.3669 (3)	0.0216 (5)	
C7	0.4218 (3)	0.22913 (11)	0.3101 (3)	0.0219 (5)	
H7	0.5122	0.2109	0.3624	0.026*	
C8	0.4721 (3)	0.09617 (12)	0.3393 (4)	0.0264 (5)	
H8A	0.4937	0.1124	0.4663	0.040*	
H8B	0.5519	0.1084	0.2671	0.040*	
H8C	0.4625	0.0482	0.3408	0.040*	
C9	0.2211 (3)	0.08562 (12)	0.1850 (4)	0.0250 (5)	
C10	0.0768 (3)	0.11842 (12)	0.1155 (4)	0.0273 (6)	
H10A	0.0953	0.1628	0.0676	0.033*	
H10B	0.0252	0.0923	0.0136	0.033*	
C11	−0.0155 (3)	0.12331 (12)	0.2724 (4)	0.0242 (5)	
C12	−0.08888 (16)	0.06244 (7)	0.3623 (2)	0.0380 (5)	0.658 (4)
H12	−0.0897	0.0174	0.3275	0.046*	0.658 (4)
S1A	−0.08888 (16)	0.06244 (7)	0.3623 (2)	0.0380 (5)	0.342 (4)
C13	−0.1560 (3)	0.09474 (17)	0.5151 (5)	0.0457 (9)	
H13	−0.2067	0.0702	0.5989	0.055*	
C14	−0.1418 (3)	0.16036 (16)	0.5300 (4)	0.0382 (7)	
H14	−0.1818	0.1853	0.6227	0.046*	
N1	0.3152 (2)	0.19186 (9)	0.2469 (3)	0.0214 (4)	
N2	0.3356 (2)	0.12514 (9)	0.2554 (3)	0.0230 (4)	
O1	0.2336 (2)	0.02600 (9)	0.1856 (3)	0.0337 (5)	
Cl1	0.67905 (7)	0.31638 (3)	0.47232 (10)	0.03056 (18)	
S1	−0.04886 (10)	0.19336 (4)	0.37322 (13)	0.0294 (3)	0.658 (4)
C12A	−0.04886 (10)	0.19336 (4)	0.37322 (13)	0.0294 (3)	0.342 (4)
H12A	−0.0232	0.2375	0.3490	0.035*	0.342 (4)

### (E)-N'-(2-Chlorophenylmethylidene)-N-methyl-2-(thiophen-2-yl)acetohydrazide (I) Atomic displacement parameters (Å^2^)

**Table:**

	*U*^11^	*U*^22^	*U*^33^	*U*^12^	*U*^13^	*U*^23^
C1	0.0228 (13)	0.0230 (11)	0.0163 (11)	0.0015 (9)	0.0041 (9)	0.0001 (8)
C2	0.0236 (13)	0.0232 (12)	0.0245 (13)	−0.0008 (9)	0.0014 (10)	−0.0001 (9)
C3	0.0240 (13)	0.0255 (12)	0.0285 (14)	0.0041 (9)	0.0020 (11)	0.0030 (10)
C4	0.0313 (14)	0.0212 (11)	0.0271 (14)	0.0010 (10)	0.0049 (11)	0.0015 (9)
C5	0.0263 (14)	0.0259 (12)	0.0231 (13)	−0.0046 (9)	0.0059 (11)	−0.0019 (9)
C6	0.0177 (12)	0.0288 (12)	0.0187 (12)	0.0019 (9)	0.0039 (10)	0.0005 (9)
C7	0.0227 (13)	0.0240 (12)	0.0196 (12)	0.0042 (9)	0.0045 (10)	0.0007 (9)
C8	0.0264 (14)	0.0255 (12)	0.0276 (14)	0.0070 (10)	0.0046 (11)	0.0036 (10)
C9	0.0274 (14)	0.0244 (12)	0.0244 (13)	0.0000 (9)	0.0080 (11)	0.0007 (9)
C10	0.0264 (14)	0.0279 (12)	0.0268 (14)	−0.0028 (10)	−0.0006 (11)	0.0007 (10)
C11	0.0180 (12)	0.0245 (12)	0.0290 (14)	−0.0007 (9)	−0.0023 (10)	0.0032 (9)
C12	0.0284 (8)	0.0372 (8)	0.0476 (10)	0.0021 (6)	0.0006 (7)	−0.0009 (6)
S1A	0.0284 (8)	0.0372 (8)	0.0476 (10)	0.0021 (6)	0.0006 (7)	−0.0009 (6)
C13	0.0185 (15)	0.058 (2)	0.060 (2)	−0.0021 (12)	0.0017 (14)	0.0294 (16)
C14	0.0324 (16)	0.0531 (18)	0.0287 (16)	0.0180 (13)	0.0023 (13)	0.0023 (12)
N1	0.0252 (11)	0.0203 (9)	0.0193 (10)	0.0035 (8)	0.0053 (8)	0.0014 (7)
N2	0.0249 (11)	0.0198 (10)	0.0249 (11)	0.0045 (8)	0.0049 (9)	0.0016 (8)
O1	0.0355 (12)	0.0219 (9)	0.0453 (12)	−0.0006 (7)	0.0115 (9)	0.0001 (8)
Cl1	0.0200 (3)	0.0360 (3)	0.0346 (4)	0.0024 (2)	−0.0017 (3)	−0.0010 (3)
S1	0.0278 (5)	0.0300 (5)	0.0302 (5)	0.0010 (3)	0.0021 (4)	−0.0037 (3)
C12A	0.0278 (5)	0.0300 (5)	0.0302 (5)	0.0010 (3)	0.0021 (4)	−0.0037 (3)

### (E)-N'-(2-Chlorophenylmethylidene)-N-methyl-2-(thiophen-2-yl)acetohydrazide (I) Geometric parameters (Å, º)

**Table:**

C1—C6	1.399 (3)	C9—N2	1.378 (3)
C1—C2	1.399 (3)	C9—C10	1.520 (4)
C1—C7	1.469 (3)	C10—C11	1.499 (4)
C2—C3	1.383 (3)	C10—H10A	0.9900
C2—H2	0.9500	C10—H10B	0.9900
C3—C4	1.391 (4)	C11—S1A	1.586 (3)
C3—H3	0.9500	C11—C12	1.586 (3)
C4—C5	1.383 (4)	C11—C12A	1.648 (3)
C4—H4	0.9500	C11—S1	1.648 (3)
C5—C6	1.388 (3)	C12—C13	1.483 (4)
C5—H5	0.9500	C12—H12	0.9500
C6—Cl1	1.748 (3)	S1A—C13	1.483 (4)
C7—N1	1.283 (3)	C13—C14	1.345 (5)
C7—H7	0.9500	C13—H13	0.9500
C8—N2	1.457 (3)	C14—C12A	1.642 (3)
C8—H8A	0.9800	C14—S1	1.642 (3)
C8—H8B	0.9800	C14—H14	0.9500
C8—H8C	0.9800	N1—N2	1.372 (3)
C9—O1	1.219 (3)	C12A—H12A	0.9500
			
C6—C1—C2	117.0 (2)	C11—C10—H10A	109.9
C6—C1—C7	122.3 (2)	C9—C10—H10A	109.9
C2—C1—C7	120.7 (2)	C11—C10—H10B	109.9
C3—C2—C1	121.6 (2)	C9—C10—H10B	109.9
C3—C2—H2	119.2	H10A—C10—H10B	108.3
C1—C2—H2	119.2	C10—C11—S1A	124.4 (2)
C2—C3—C4	119.9 (2)	C10—C11—C12	124.4 (2)
C2—C3—H3	120.0	C10—C11—C12A	123.04 (19)
C4—C3—H3	120.0	S1A—C11—C12A	112.59 (17)
C5—C4—C3	120.1 (2)	C10—C11—S1	123.04 (19)
C5—C4—H4	119.9	C12—C11—S1	112.59 (17)
C3—C4—H4	119.9	C13—C12—C11	101.16 (18)
C4—C5—C6	119.2 (2)	C13—C12—H12	129.4
C4—C5—H5	120.4	C11—C12—H12	129.4
C6—C5—H5	120.4	C13—S1A—C11	101.16 (18)
C5—C6—C1	122.2 (2)	C14—C13—C12	117.1 (3)
C5—C6—Cl1	117.13 (19)	C14—C13—S1A	117.1 (3)
C1—C6—Cl1	120.61 (18)	C14—C13—H13	121.4
N1—C7—C1	118.5 (2)	C12—C13—H13	121.4
N1—C7—H7	120.7	C13—C14—C12A	113.8 (2)
C1—C7—H7	120.7	C13—C14—S1	113.8 (2)
N2—C8—H8A	109.5	C13—C14—H14	123.1
N2—C8—H8B	109.5	S1—C14—H14	123.1
H8A—C8—H8B	109.5	C7—N1—N2	118.3 (2)
N2—C8—H8C	109.5	N1—N2—C9	117.8 (2)
H8A—C8—H8C	109.5	N1—N2—C8	121.8 (2)
H8B—C8—H8C	109.5	C9—N2—C8	120.4 (2)
O1—C9—N2	120.8 (2)	C14—S1—C11	95.27 (15)
O1—C9—C10	121.1 (2)	C14—C12A—C11	95.27 (15)
N2—C9—C10	118.0 (2)	C14—C12A—H12A	132.4
C11—C10—C9	108.8 (2)	C11—C12A—H12A	132.4
			
C6—C1—C2—C3	−0.3 (4)	S1—C11—C12—C13	−2.6 (2)
C7—C1—C2—C3	179.6 (2)	C10—C11—S1A—C13	176.2 (2)
C1—C2—C3—C4	0.0 (4)	C12A—C11—S1A—C13	−2.6 (2)
C2—C3—C4—C5	0.8 (4)	C11—C12—C13—C14	2.0 (3)
C3—C4—C5—C6	−1.2 (4)	C11—S1A—C13—C14	2.0 (3)
C4—C5—C6—C1	1.0 (4)	S1A—C13—C14—C12A	−0.6 (3)
C4—C5—C6—Cl1	−177.7 (2)	C12—C13—C14—S1	−0.6 (3)
C2—C1—C6—C5	−0.2 (4)	C1—C7—N1—N2	179.5 (2)
C7—C1—C6—C5	179.9 (2)	C7—N1—N2—C9	−179.2 (2)
C2—C1—C6—Cl1	178.47 (18)	C7—N1—N2—C8	2.1 (3)
C7—C1—C6—Cl1	−1.5 (3)	O1—C9—N2—N1	178.6 (2)
C6—C1—C7—N1	179.4 (2)	C10—C9—N2—N1	−4.2 (3)
C2—C1—C7—N1	−0.5 (4)	O1—C9—N2—C8	−2.7 (4)
O1—C9—C10—C11	87.0 (3)	C10—C9—N2—C8	174.4 (2)
N2—C9—C10—C11	−90.1 (3)	C13—C14—S1—C11	−1.0 (3)
C9—C10—C11—S1A	−70.0 (3)	C10—C11—S1—C14	−176.6 (2)
C9—C10—C11—C12	−70.0 (3)	C12—C11—S1—C14	2.24 (19)
C9—C10—C11—C12A	108.7 (2)	C13—C14—C12A—C11	−1.0 (3)
C9—C10—C11—S1	108.7 (2)	C10—C11—C12A—C14	−176.6 (2)
C10—C11—C12—C13	176.2 (2)	S1A—C11—C12A—C14	2.24 (19)

### (E)-N'-(2-Chlorophenylmethylidene)-N-methyl-2-(thiophen-2-yl)acetohydrazide (I) Hydrogen-bond geometry (Å, º)

Cg1 is the centroid of the thiophene ring, Cg2 is the centroid of the benzene ring.

**Table:**

*D*—H···*A*	*D*—H	H···*A*	*D*···*A*	*D*—H···*A*
C13—H13···O1^i^	0.95	2.54	3.410 (4)	153
C3—H3···*Cg*1^ii^	0.95	2.83	3.612 (3)	140
C8—H8*A*···*Cg*2^iii^	0.98	2.71	3.544 (3)	144

Symmetry codes: (i) −*x*, −*y*, −*z*+1; (ii) *x*, −*y*+1/2, *z*−1/2; (iii) *x*, −*y*+1/2, *z*+1/2.

### (II) Crystal data

**Table:**

C_14_H_13_BrN_2_OS	*F*(000) = 680
*M**_r_* = 337.23	*D*_x_ = 1.628 Mg m^−^^3^
Monoclinic, *P*2_1_/*c*	Mo *K*α radiation, λ = 0.71073 Å
*a* = 9.4479 (7) Å	Cell parameters from 14004 reflections
*b* = 20.2175 (14) Å	θ = 2.4–27.5°
*c* = 7.2552 (5) Å	µ = 3.13 mm^−^^1^
β = 96.9343 (13)°	*T* = 100 K
*V* = 1375.70 (17) Å^3^	Rod, light brown
*Z* = 4	0.26 × 0.06 × 0.05 mm

### (II) Data collection

**Table:**

Rigaku Mercury CCD diffractometer	2897 reflections with *I* > 2σ(*I*)
ω scans	*R*_int_ = 0.040
Absorption correction: multi-scan (SADABS; Bruker, 2004)	θ_max_ = 27.5°, θ_min_ = 2.4°
*T*_min_ = 0.756, *T*_max_ = 1.000	*h* = −12→11
14778 measured reflections	*k* = −26→26
3163 independent reflections	*l* = −8→9

### (II) Refinement

**Table:**

Refinement on *F*^2^	Primary atom site location: structure-invariant direct methods
Least-squares matrix: full	Hydrogen site location: inferred from neighbouring sites
*R*[*F*^2^ > 2σ(*F*^2^)] = 0.025	H-atom parameters constrained
*wR*(*F*^2^) = 0.064	*w* = 1/[σ^2^(*F*_o_^2^) + (0.029*P*)^2^ + 0.8865*P*] where *P* = (*F*_o_^2^ + 2*F*_c_^2^)/3
*S* = 1.06	(Δ/σ)_max_ = 0.001
3163 reflections	Δρ_max_ = 0.48 e Å^−^^3^
174 parameters	Δρ_min_ = −0.43 e Å^−^^3^
0 restraints	

### (II) Special details

**Table:**

Geometry. All esds (except the esd in the dihedral angle between two l.s. planes) are estimated using the full covariance matrix. The cell esds are taken into account individually in the estimation of esds in distances, angles and torsion angles; correlations between esds in cell parameters are only used when they are defined by crystal symmetry. An approximate (isotropic) treatment of cell esds is used for estimating esds involving l.s. planes.

### (II) Fractional atomic coordinates and isotropic or equivalent isotropic displacement parameters (Å^2^)

**Table:**

	*x*	*y*	*z*	*U*_iso_*/*U*_eq_	Occ. (<1)
C1	0.09809 (17)	0.30257 (8)	0.1963 (2)	0.0165 (3)	
C2	0.22907 (18)	0.32858 (8)	0.2763 (2)	0.0187 (3)	
H2	0.3031	0.2991	0.3235	0.022*	
C3	0.25292 (18)	0.39601 (9)	0.2878 (2)	0.0206 (3)	
H3	0.3425	0.4124	0.3426	0.025*	
C4	0.14614 (19)	0.43993 (8)	0.2194 (2)	0.0207 (3)	
H4	0.1633	0.4862	0.2257	0.025*	
C5	0.01471 (18)	0.41630 (8)	0.1420 (2)	0.0193 (3)	
H5	−0.0594	0.4461	0.0973	0.023*	
C6	−0.00727 (17)	0.34818 (9)	0.1306 (2)	0.0173 (3)	
C7	0.07642 (17)	0.23064 (8)	0.1846 (2)	0.0171 (3)	
H7	−0.0121	0.2128	0.1309	0.020*	
C8	0.02550 (18)	0.09658 (9)	0.1503 (3)	0.0208 (3)	
H8A	0.0048	0.1134	0.0232	0.031*	
H8B	−0.0523	0.1087	0.2217	0.031*	
H8C	0.0345	0.0483	0.1475	0.031*	
C9	0.26952 (19)	0.08518 (9)	0.3080 (2)	0.0203 (3)	
C10	0.41014 (18)	0.11767 (9)	0.3834 (3)	0.0218 (4)	
H10A	0.3923	0.1624	0.4309	0.026*	
H10B	0.4585	0.0911	0.4870	0.026*	
C11	0.50282 (17)	0.12232 (8)	0.2296 (3)	0.0199 (3)	
C12	0.57818 (11)	0.06159 (5)	0.14775 (16)	0.0320 (3)	0.677 (3)
H12	0.5796	0.0166	0.1856	0.038*	0.677 (3)
S1A	0.57818 (11)	0.06159 (5)	0.14775 (16)	0.0320 (3)	0.323 (3)
C13	0.6464 (2)	0.09279 (12)	−0.0038 (3)	0.0368 (5)	
H13	0.6988	0.0675	−0.0828	0.044*	
C14	0.6306 (2)	0.15886 (11)	−0.0243 (3)	0.0316 (4)	
H14	0.6708	0.1834	−0.1168	0.038*	
N1	0.17897 (15)	0.19243 (7)	0.2481 (2)	0.0171 (3)	
N2	0.15849 (15)	0.12529 (7)	0.2374 (2)	0.0177 (3)	
O1	0.25756 (14)	0.02499 (6)	0.3042 (2)	0.0272 (3)	
S1	0.53516 (6)	0.19288 (3)	0.12608 (9)	0.02306 (19)	0.677 (3)
C12A	0.53516 (6)	0.19288 (3)	0.12608 (9)	0.02306 (19)	0.323 (3)
H12A	0.5082	0.2373	0.1464	0.028*	0.323 (3)
Br1	−0.18904 (2)	0.31960 (2)	0.01542 (3)	0.02297 (7)	

### (II) Atomic displacement parameters (Å^2^)

**Table:**

	*U*^11^	*U*^22^	*U*^33^	*U*^12^	*U*^13^	*U*^23^
C1	0.0183 (8)	0.0179 (8)	0.0135 (8)	−0.0010 (6)	0.0025 (6)	−0.0001 (6)
C2	0.0183 (8)	0.0192 (8)	0.0179 (8)	0.0008 (6)	−0.0001 (6)	0.0008 (6)
C3	0.0201 (8)	0.0207 (8)	0.0206 (9)	−0.0030 (6)	0.0012 (7)	−0.0018 (6)
C4	0.0256 (8)	0.0163 (8)	0.0207 (9)	−0.0019 (6)	0.0050 (7)	−0.0011 (6)
C5	0.0213 (8)	0.0189 (8)	0.0181 (9)	0.0042 (6)	0.0043 (6)	0.0018 (6)
C6	0.0164 (7)	0.0215 (8)	0.0140 (8)	−0.0010 (6)	0.0019 (6)	0.0004 (6)
C7	0.0185 (7)	0.0183 (8)	0.0145 (8)	−0.0031 (6)	0.0021 (6)	−0.0008 (6)
C8	0.0203 (8)	0.0184 (8)	0.0239 (9)	−0.0048 (6)	0.0038 (7)	−0.0028 (7)
C9	0.0236 (8)	0.0192 (8)	0.0191 (9)	0.0005 (7)	0.0074 (7)	−0.0006 (6)
C10	0.0219 (8)	0.0211 (8)	0.0219 (9)	0.0030 (7)	0.0002 (7)	−0.0001 (7)
C11	0.0150 (7)	0.0200 (8)	0.0236 (9)	0.0010 (6)	−0.0020 (6)	−0.0026 (7)
C12	0.0259 (5)	0.0317 (6)	0.0370 (6)	−0.0031 (4)	−0.0013 (4)	0.0004 (4)
S1A	0.0259 (5)	0.0317 (6)	0.0370 (6)	−0.0031 (4)	−0.0013 (4)	0.0004 (4)
C13	0.0170 (8)	0.0462 (13)	0.0465 (14)	0.0014 (8)	0.0012 (8)	−0.0239 (10)
C14	0.0305 (10)	0.0408 (12)	0.0240 (10)	−0.0174 (9)	0.0047 (8)	−0.0038 (8)
N1	0.0208 (7)	0.0141 (7)	0.0168 (7)	−0.0024 (5)	0.0043 (6)	−0.0013 (5)
N2	0.0196 (7)	0.0132 (6)	0.0205 (7)	−0.0027 (5)	0.0036 (6)	−0.0017 (5)
O1	0.0291 (7)	0.0167 (6)	0.0371 (8)	0.0010 (5)	0.0099 (6)	0.0005 (5)
S1	0.0217 (3)	0.0234 (3)	0.0237 (3)	−0.0004 (2)	0.0013 (2)	0.0023 (2)
C12A	0.0217 (3)	0.0234 (3)	0.0237 (3)	−0.0004 (2)	0.0013 (2)	0.0023 (2)
Br1	0.01578 (10)	0.02638 (11)	0.02587 (12)	−0.00184 (6)	−0.00109 (7)	0.00178 (7)

### (II) Geometric parameters (Å, º)

**Table:**

C1—C6	1.398 (2)	C9—N2	1.375 (2)
C1—C2	1.404 (2)	C9—C10	1.523 (2)
C1—C7	1.470 (2)	C10—C11	1.502 (2)
C2—C3	1.383 (2)	C10—H10A	0.9900
C2—H2	0.9500	C10—H10B	0.9900
C3—C4	1.390 (2)	C11—S1A	1.571 (2)
C3—H3	0.9500	C11—C12	1.571 (2)
C4—C5	1.385 (2)	C11—C12A	1.6576 (19)
C4—H4	0.9500	C11—S1	1.6576 (19)
C5—C6	1.394 (2)	C12—C13	1.481 (3)
C5—H5	0.9500	C12—H12	0.9500
C6—Br1	1.9064 (16)	S1A—C13	1.481 (3)
C7—N1	1.281 (2)	C13—C14	1.350 (3)
C7—H7	0.9500	C13—H13	0.9500
C8—N2	1.457 (2)	C14—C12A	1.648 (2)
C8—H8A	0.9800	C14—S1	1.648 (2)
C8—H8B	0.9800	C14—H14	0.9500
C8—H8C	0.9800	N1—N2	1.3720 (19)
C9—O1	1.222 (2)	C12A—H12A	0.9500
			
C6—C1—C2	116.73 (15)	C11—C10—H10A	109.9
C6—C1—C7	122.98 (15)	C9—C10—H10A	109.9
C2—C1—C7	120.28 (15)	C11—C10—H10B	109.9
C3—C2—C1	121.56 (16)	C9—C10—H10B	109.9
C3—C2—H2	119.2	H10A—C10—H10B	108.3
C1—C2—H2	119.2	C10—C11—S1A	124.27 (14)
C2—C3—C4	120.15 (16)	C10—C11—C12	124.27 (14)
C2—C3—H3	119.9	C10—C11—C12A	123.11 (13)
C4—C3—H3	119.9	S1A—C11—C12A	112.62 (12)
C5—C4—C3	120.09 (16)	C10—C11—S1	123.11 (13)
C5—C4—H4	120.0	C12—C11—S1	112.62 (12)
C3—C4—H4	120.0	C13—C12—C11	101.85 (12)
C4—C5—C6	119.01 (15)	C13—C12—H12	129.1
C4—C5—H5	120.5	C11—C12—H12	129.1
C6—C5—H5	120.5	C13—S1A—C11	101.85 (12)
C5—C6—C1	122.46 (15)	C14—C13—C12	116.81 (17)
C5—C6—Br1	116.44 (12)	C14—C13—S1A	116.81 (17)
C1—C6—Br1	121.09 (13)	C14—C13—H13	121.6
N1—C7—C1	118.82 (15)	C12—C13—H13	121.6
N1—C7—H7	120.6	C13—C14—C12A	113.69 (17)
C1—C7—H7	120.6	C13—C14—S1	113.69 (17)
N2—C8—H8A	109.5	C13—C14—H14	123.2
N2—C8—H8B	109.5	S1—C14—H14	123.2
H8A—C8—H8B	109.5	C7—N1—N2	118.76 (14)
N2—C8—H8C	109.5	N1—N2—C9	117.80 (14)
H8A—C8—H8C	109.5	N1—N2—C8	121.80 (14)
H8B—C8—H8C	109.5	C9—N2—C8	120.38 (14)
O1—C9—N2	120.96 (16)	C14—S1—C11	94.95 (10)
O1—C9—C10	120.78 (16)	C14—C12A—C11	94.95 (10)
N2—C9—C10	118.20 (15)	C14—C12A—H12A	132.5
C11—C10—C9	108.77 (14)	C11—C12A—H12A	132.5
			
C6—C1—C2—C3	−0.5 (3)	S1—C11—C12—C13	−2.72 (14)
C7—C1—C2—C3	179.45 (16)	C10—C11—S1A—C13	176.72 (15)
C1—C2—C3—C4	0.0 (3)	C12A—C11—S1A—C13	−2.72 (14)
C2—C3—C4—C5	1.0 (3)	C11—C12—C13—C14	1.9 (2)
C3—C4—C5—C6	−1.3 (3)	C11—S1A—C13—C14	1.9 (2)
C4—C5—C6—C1	0.7 (3)	S1A—C13—C14—C12A	−0.4 (2)
C4—C5—C6—Br1	−178.15 (13)	C12—C13—C14—S1	−0.4 (2)
C2—C1—C6—C5	0.2 (2)	C1—C7—N1—N2	179.85 (14)
C7—C1—C6—C5	−179.79 (16)	C7—N1—N2—C9	−179.36 (15)
C2—C1—C6—Br1	179.02 (12)	C7—N1—N2—C8	2.2 (2)
C7—C1—C6—Br1	−1.0 (2)	O1—C9—N2—N1	179.36 (16)
C6—C1—C7—N1	179.85 (16)	C10—C9—N2—N1	−3.3 (2)
C2—C1—C7—N1	−0.1 (2)	O1—C9—N2—C8	−2.2 (2)
O1—C9—C10—C11	85.9 (2)	C10—C9—N2—C8	175.12 (15)
N2—C9—C10—C11	−91.44 (18)	C13—C14—S1—C11	−1.24 (17)
C9—C10—C11—S1A	−72.27 (19)	C10—C11—S1—C14	−177.05 (15)
C9—C10—C11—C12	−72.27 (19)	C12—C11—S1—C14	2.39 (13)
C9—C10—C11—C12A	107.11 (16)	C13—C14—C12A—C11	−1.24 (17)
C9—C10—C11—S1	107.11 (16)	C10—C11—C12A—C14	−177.05 (15)
C10—C11—C12—C13	176.72 (15)	S1A—C11—C12A—C14	2.39 (13)

### (II) Hydrogen-bond geometry (Å, º)

Cg1 is the centroid of the thiophene ring, Cg2 is the centroid of the benzene ring.

**Table:**

*D*—H···*A*	*D*—H	H···*A*	*D*···*A*	*D*—H···*A*
C13—H13···O1^i^	0.95	2.53	3.424 (2)	156
C3—H3···*Cg*1^ii^	0.95	2.82	3.6021 (18)	141
C8—H8*A*···*Cg*2^iii^	0.98	2.67	3.495 (2)	142

Symmetry codes: (i) −*x*+1, −*y*, −*z*; (ii) *x*, −*y*+1/2, *z*−1/2; (iii) *x*, −*y*+1/2, *z*+1/2.

### (III) Crystal data

**Table:**

C_14_H_13_ClN_2_OS	*F*(000) = 608
*M**_r_* = 292.77	*D*_x_ = 1.415 Mg m^−^^3^
Monoclinic, *P*2_1_/*n*	Mo *K*α radiation, λ = 0.71075 Å
*a* = 4.2194 (2) Å	Cell parameters from 9584 reflections
*b* = 13.0131 (9) Å	θ = 2.9–27.5°
*c* = 25.0758 (18) Å	µ = 0.42 mm^−^^1^
β = 93.752 (4)°	*T* = 100 K
*V* = 1373.90 (15) Å^3^	Needle, colourless
*Z* = 4	0.22 × 0.01 × 0.01 mm

### (III) Data collection

**Table:**

Rigaku Mercury CCD diffractometer	1766 reflections with *I* > 2σ(*I*)
ω scans	*R*_int_ = 0.127
Absorption correction: multi-scan (SADABS; Bruker, 2004)	θ_max_ = 27.4°, θ_min_ = 2.9°
*T*_min_ = 0.615, *T*_max_ = 1.000	*h* = −5→5
12868 measured reflections	*k* = −16→16
3121 independent reflections	*l* = −32→32

### (III) Refinement

**Table:**

Refinement on *F*^2^	Primary atom site location: structure-invariant direct methods
Least-squares matrix: full	Hydrogen site location: mixed
*R*[*F*^2^ > 2σ(*F*^2^)] = 0.116	H-atom parameters constrained
*wR*(*F*^2^) = 0.297	*w* = 1/[σ^2^(*F*_o_^2^) + (0.0832*P*)^2^ + 8.0037*P*] where *P* = (*F*_o_^2^ + 2*F*_c_^2^)/3
*S* = 1.16	(Δ/σ)_max_ = 0.006
3121 reflections	Δρ_max_ = 1.00 e Å^−^^3^
184 parameters	Δρ_min_ = −0.45 e Å^−^^3^
0 restraints	

### (III) Special details

**Table:**

Geometry. All esds (except the esd in the dihedral angle between two l.s. planes) are estimated using the full covariance matrix. The cell esds are taken into account individually in the estimation of esds in distances, angles and torsion angles; correlations between esds in cell parameters are only used when they are defined by crystal symmetry. An approximate (isotropic) treatment of cell esds is used for estimating esds involving l.s. planes.

### (III) Fractional atomic coordinates and isotropic or equivalent isotropic displacement parameters (Å^2^)

**Table:**

	*x*	*y*	*z*	*U*_iso_*/*U*_eq_	Occ. (<1)
C1	0.2176 (17)	0.2480 (6)	0.1409 (2)	0.0278 (15)	
C2	0.0167 (16)	0.2955 (6)	0.1012 (3)	0.0281 (15)	
H2	−0.0133	0.3679	0.1017	0.034*	
C3	−0.1373 (17)	0.2366 (6)	0.0613 (3)	0.0339 (18)	
H3	−0.2716	0.2690	0.0344	0.041*	
C4	−0.0966 (18)	0.1304 (6)	0.0603 (3)	0.0352 (17)	
H4	−0.2041	0.0901	0.0332	0.042*	0.854 (7)
C5	0.098 (2)	0.0838 (6)	0.0994 (3)	0.0383 (18)	
H5	0.1284	0.0115	0.0987	0.046*	0.146 (7)
C6	0.2538 (18)	0.1414 (6)	0.1398 (3)	0.0322 (17)	
H6	0.3852	0.1082	0.1668	0.039*	
C7	0.3910 (16)	0.3074 (5)	0.1837 (3)	0.0258 (15)	
H7	0.5213	0.2733	0.2105	0.031*	
C8	0.7209 (18)	0.4074 (6)	0.2680 (3)	0.0335 (17)	
H8A	0.5903	0.3557	0.2847	0.050*	
H8B	0.7949	0.4582	0.2949	0.050*	
H8C	0.9042	0.3739	0.2534	0.050*	
C9	0.5187 (17)	0.5634 (6)	0.2228 (3)	0.0307 (17)	
C10	0.3000 (17)	0.6118 (6)	0.1778 (3)	0.0324 (17)	
H10A	0.1189	0.5651	0.1691	0.039*	
H10B	0.2137	0.6774	0.1907	0.039*	
C11	0.4715 (15)	0.6322 (5)	0.1277 (2)	0.0245 (14)	
C12	0.5901 (13)	0.7328 (5)	0.11266 (19)	0.048 (2)	0.795 (9)
H12	0.5807	0.7956	0.1319	0.058*	0.795 (9)
S1A	0.5901 (13)	0.7328 (5)	0.11266 (19)	0.048 (2)	0.205 (9)
C13	0.7313 (18)	0.7147 (6)	0.0596 (3)	0.0353 (17)	
H13	0.8266	0.7681	0.0404	0.042*	
C14	0.7127 (17)	0.6182 (6)	0.0416 (3)	0.0342 (17)	
H14	0.7921	0.5975	0.0087	0.041*	
N1	0.3638 (13)	0.4055 (4)	0.1843 (2)	0.0250 (13)	
N2	0.5314 (14)	0.4588 (5)	0.2249 (2)	0.0302 (13)	
O1	0.6759 (12)	0.6183 (4)	0.25450 (18)	0.0362 (13)	
Cl1	0.1534 (7)	−0.04694 (19)	0.09763 (10)	0.0537 (9)	0.854 (7)
Cl2	−0.296 (5)	0.0407 (14)	0.0203 (7)	0.067 (7)	0.146 (7)
S1	0.5363 (5)	0.53654 (16)	0.08241 (8)	0.0261 (7)	0.795 (9)
C12A	0.5363 (5)	0.53654 (16)	0.08241 (8)	0.0261 (7)	0.205 (9)
H12A	0.4903	0.4651	0.0810	0.031*	0.205 (9)

### (III) Atomic displacement parameters (Å^2^)

**Table:**

	*U*^11^	*U*^22^	*U*^33^	*U*^12^	*U*^13^	*U*^23^
C1	0.035 (4)	0.031 (4)	0.018 (3)	0.001 (3)	0.007 (3)	0.000 (3)
C2	0.026 (4)	0.026 (4)	0.033 (4)	0.000 (3)	0.008 (3)	0.001 (3)
C3	0.025 (4)	0.053 (5)	0.024 (3)	−0.002 (3)	0.001 (3)	0.000 (3)
C4	0.032 (4)	0.035 (4)	0.038 (4)	−0.006 (3)	−0.001 (3)	−0.010 (4)
C5	0.046 (5)	0.030 (4)	0.039 (4)	−0.002 (4)	0.001 (3)	−0.007 (3)
C6	0.042 (4)	0.028 (4)	0.027 (3)	0.000 (3)	0.003 (3)	0.001 (3)
C7	0.027 (4)	0.029 (4)	0.022 (3)	0.003 (3)	0.002 (3)	0.001 (3)
C8	0.041 (4)	0.035 (4)	0.024 (3)	−0.008 (3)	−0.003 (3)	0.004 (3)
C9	0.029 (4)	0.045 (5)	0.019 (3)	−0.004 (3)	0.007 (3)	−0.005 (3)
C10	0.030 (4)	0.035 (4)	0.032 (4)	0.004 (3)	0.002 (3)	−0.008 (3)
C11	0.024 (3)	0.025 (4)	0.024 (3)	0.005 (3)	−0.004 (3)	−0.001 (3)
C12	0.051 (4)	0.063 (4)	0.029 (3)	−0.004 (3)	−0.008 (2)	0.013 (2)
S1A	0.051 (4)	0.063 (4)	0.029 (3)	−0.004 (3)	−0.008 (2)	0.013 (2)
C13	0.041 (4)	0.035 (4)	0.029 (4)	−0.003 (4)	−0.007 (3)	0.008 (3)
C14	0.032 (4)	0.045 (5)	0.024 (3)	−0.001 (3)	−0.007 (3)	0.004 (3)
N1	0.030 (3)	0.028 (3)	0.017 (2)	−0.001 (2)	0.001 (2)	−0.005 (2)
N2	0.035 (3)	0.034 (3)	0.020 (3)	−0.001 (3)	−0.003 (2)	−0.004 (3)
O1	0.047 (3)	0.042 (3)	0.020 (2)	−0.005 (3)	0.003 (2)	−0.008 (2)
Cl1	0.085 (2)	0.0220 (12)	0.0519 (15)	−0.0009 (12)	−0.0112 (13)	−0.0015 (11)
Cl2	0.086 (13)	0.059 (11)	0.059 (10)	−0.023 (9)	0.028 (9)	−0.035 (9)
S1	0.0310 (12)	0.0240 (11)	0.0228 (10)	0.0019 (9)	−0.0021 (7)	−0.0015 (8)
C12A	0.0310 (12)	0.0240 (11)	0.0228 (10)	0.0019 (9)	−0.0021 (7)	−0.0015 (8)

### (III) Geometric parameters (Å, º)

**Table:**

C1—C6	1.395 (10)	C9—C10	1.545 (10)
C1—C2	1.408 (10)	C10—C11	1.514 (9)
C1—C7	1.478 (10)	C10—H10A	0.9900
C2—C3	1.388 (10)	C10—H10B	0.9900
C2—H2	0.9500	C11—S1A	1.461 (9)
C3—C4	1.394 (11)	C11—C12	1.461 (9)
C3—H3	0.9500	C11—C12A	1.718 (7)
C4—C5	1.377 (11)	C11—S1	1.718 (7)
C4—Cl2	1.724 (17)	C12—C13	1.511 (9)
C4—H4	0.9500	C12—H12	0.9500
C5—C6	1.392 (10)	S1A—C13	1.511 (9)
C5—Cl1	1.718 (8)	C13—C14	1.334 (11)
C5—H5	0.9500	C13—H13	0.9500
C6—H6	0.9500	C14—C12A	1.682 (8)
C7—N1	1.281 (9)	C14—S1	1.682 (8)
C7—H7	0.9500	C14—H14	0.9500
C8—N2	1.464 (9)	N1—N2	1.387 (8)
C8—H8A	0.9800	Cl1—H5	0.7679
C8—H8B	0.9800	Cl2—Cl2^i^	2.21 (3)
C8—H8C	0.9800	Cl2—H4	0.8086
C9—O1	1.229 (8)	C12A—H12A	0.9500
C9—N2	1.364 (10)		
			
C6—C1—C2	119.0 (7)	C11—C10—C9	112.5 (6)
C6—C1—C7	119.0 (7)	C11—C10—H10A	109.1
C2—C1—C7	122.0 (7)	C9—C10—H10A	109.1
C3—C2—C1	119.9 (7)	C11—C10—H10B	109.1
C3—C2—H2	120.0	C9—C10—H10B	109.1
C1—C2—H2	120.0	H10A—C10—H10B	107.8
C2—C3—C4	120.5 (7)	S1A—C11—C10	124.1 (6)
C2—C3—H3	119.7	C12—C11—C10	124.1 (6)
C4—C3—H3	119.7	S1A—C11—C12A	114.0 (5)
C5—C4—C3	119.5 (7)	C10—C11—C12A	121.8 (5)
C5—C4—Cl2	111.2 (9)	C12—C11—S1	114.0 (5)
C3—C4—Cl2	128.8 (10)	C10—C11—S1	121.8 (5)
C5—C4—H4	120.0	C11—C12—C13	104.4 (6)
C3—C4—H4	120.4	C11—C12—H12	127.8
Cl2—C4—H4	10.5	C13—C12—H12	127.8
C4—C5—C6	120.8 (7)	C11—S1A—C13	104.4 (6)
C4—C5—Cl1	119.6 (6)	C14—C13—C12	115.3 (7)
C6—C5—Cl1	119.6 (6)	C14—C13—S1A	115.3 (7)
C4—C5—H5	120.0	C14—C13—H13	122.4
C6—C5—H5	119.2	C12—C13—H13	122.4
Cl1—C5—H5	0.5	C13—C14—C12A	114.1 (6)
C5—C6—C1	120.2 (7)	C13—C14—S1	114.1 (6)
C5—C6—H6	119.9	C13—C14—H14	123.0
C1—C6—H6	119.9	S1—C14—H14	123.0
N1—C7—C1	119.3 (6)	C7—N1—N2	117.7 (6)
N1—C7—H7	120.3	C9—N2—N1	117.0 (6)
C1—C7—H7	120.3	C9—N2—C8	120.2 (6)
N2—C8—H8A	109.5	N1—N2—C8	122.7 (6)
N2—C8—H8B	109.5	C5—Cl1—H5	0.6
H8A—C8—H8B	109.5	C4—Cl2—Cl2^i^	158.2 (15)
N2—C8—H8C	109.5	C4—Cl2—H4	12.4
H8A—C8—H8C	109.5	Cl2^i^—Cl2—H4	154.9
H8B—C8—H8C	109.5	C14—S1—C11	92.3 (4)
O1—C9—N2	122.5 (7)	C14—C12A—C11	92.3 (4)
O1—C9—C10	120.5 (7)	C14—C12A—H12A	133.9
N2—C9—C10	117.1 (6)	C11—C12A—H12A	133.9
			
C6—C1—C2—C3	−1.1 (10)	S1—C11—C12—C13	0.5 (6)
C7—C1—C2—C3	179.0 (6)	C10—C11—S1A—C13	−178.3 (6)
C1—C2—C3—C4	0.4 (10)	C12A—C11—S1A—C13	0.5 (6)
C2—C3—C4—C5	0.1 (11)	C11—C12—C13—C14	−0.1 (8)
C2—C3—C4—Cl2	171.4 (9)	C11—S1A—C13—C14	−0.1 (8)
C3—C4—C5—C6	0.1 (12)	S1A—C13—C14—C12A	−0.3 (8)
Cl2—C4—C5—C6	−172.7 (8)	C12—C13—C14—S1	−0.3 (8)
C3—C4—C5—Cl1	−179.0 (6)	C1—C7—N1—N2	−179.6 (6)
Cl2—C4—C5—Cl1	8.3 (10)	O1—C9—N2—N1	−174.7 (6)
C4—C5—C6—C1	−0.8 (11)	C10—C9—N2—N1	4.3 (9)
Cl1—C5—C6—C1	178.3 (6)	O1—C9—N2—C8	4.1 (10)
C2—C1—C6—C5	1.3 (10)	C10—C9—N2—C8	−176.9 (6)
C7—C1—C6—C5	−178.8 (7)	C7—N1—N2—C9	175.3 (6)
C6—C1—C7—N1	178.6 (6)	C7—N1—N2—C8	−3.5 (9)
C2—C1—C7—N1	−1.5 (10)	C5—C4—Cl2—Cl2^i^	123 (4)
O1—C9—C10—C11	88.1 (8)	C3—C4—Cl2—Cl2^i^	−49 (5)
N2—C9—C10—C11	−90.9 (8)	C13—C14—S1—C11	0.6 (6)
C9—C10—C11—S1A	−100.6 (8)	C12—C11—S1—C14	−0.6 (5)
C9—C10—C11—C12	−100.6 (8)	C10—C11—S1—C14	178.3 (6)
C9—C10—C11—C12A	80.6 (7)	C13—C14—C12A—C11	0.6 (6)
C9—C10—C11—S1	80.6 (7)	S1A—C11—C12A—C14	−0.6 (5)
C10—C11—C12—C13	−178.3 (6)	C10—C11—C12A—C14	178.3 (6)

Symmetry code: (i) −*x*−1, −*y*, −*z*.

### (III) Hydrogen-bond geometry (Å, º)

**Table:**

*D*—H···*A*	*D*—H	H···*A*	*D*···*A*	*D*—H···*A*
C6—H6···O1^ii^	0.95	2.62	3.474 (9)	150
C7—H7···O1^ii^	0.95	2.52	3.381 (9)	152
C12—H12···Cl1^iii^	0.95	2.83	3.415 (7)	121

Symmetry codes: (ii) −*x*+3/2, *y*−1/2, −*z*+1/2; (iii) *x*, *y*+1, *z*.

### (IV) Crystal data

**Table:**

C_14_H_13_ClN_2_OS	*F*(000) = 608
*M**_r_* = 292.77	*D*_x_ = 1.410 Mg m^−^^3^
Monoclinic, *P*2_1_/*n*	Mo *K*α radiation, λ = 0.71073 Å
*a* = 6.7454 (5) Å	Cell parameters from 14502 reflections
*b* = 20.2993 (14) Å	θ = 2.3–27.5°
*c* = 10.1592 (7) Å	µ = 0.42 mm^−^^1^
β = 97.510 (2)°	*T* = 100 K
*V* = 1379.14 (17) Å^3^	Block, colourless
*Z* = 4	0.30 × 0.17 × 0.10 mm

### (IV) Data collection

**Table:**

Rigaku Mercury CCD diffractometer	2929 reflections with *I* > 2σ(*I*)
ω scans	*R*_int_ = 0.033
Absorption correction: multi-scan (SADABS; Bruker, 2004)	θ_max_ = 27.5°, θ_min_ = 2.9°
*T*_min_ = 0.843, *T*_max_ = 1.000	*h* = −7→8
14763 measured reflections	*k* = −25→26
3165 independent reflections	*l* = −12→13

### (IV) Refinement

**Table:**

Refinement on *F*^2^	Primary atom site location: structure-invariant direct methods
Least-squares matrix: full	Hydrogen site location: inferred from neighbouring sites
*R*[*F*^2^ > 2σ(*F*^2^)] = 0.033	H-atom parameters constrained
*wR*(*F*^2^) = 0.091	*w* = 1/[σ^2^(*F*_o_^2^) + (0.0494*P*)^2^ + 0.4663*P*] where *P* = (*F*_o_^2^ + 2*F*_c_^2^)/3
*S* = 1.05	(Δ/σ)_max_ < 0.001
3165 reflections	Δρ_max_ = 0.53 e Å^−^^3^
174 parameters	Δρ_min_ = −0.40 e Å^−^^3^
0 restraints	

### (IV) Special details

**Table:**

Geometry. All esds (except the esd in the dihedral angle between two l.s. planes) are estimated using the full covariance matrix. The cell esds are taken into account individually in the estimation of esds in distances, angles and torsion angles; correlations between esds in cell parameters are only used when they are defined by crystal symmetry. An approximate (isotropic) treatment of cell esds is used for estimating esds involving l.s. planes.

### (IV) Fractional atomic coordinates and isotropic or equivalent isotropic displacement parameters (Å^2^)

**Table:**

	*x*	*y*	*z*	*U*_iso_*/*U*_eq_	Occ. (<1)
C1	0.30559 (18)	0.18484 (6)	0.17665 (11)	0.0232 (2)	
C2	0.29278 (19)	0.11619 (6)	0.16729 (12)	0.0258 (3)	
H2	0.4040	0.0899	0.2020	0.031*	
C3	0.1195 (2)	0.08611 (6)	0.10784 (13)	0.0277 (3)	
H3	0.1109	0.0395	0.1019	0.033*	
C4	−0.04180 (18)	0.12533 (6)	0.05692 (12)	0.0251 (2)	
C5	−0.03348 (19)	0.19335 (7)	0.06381 (12)	0.0266 (3)	
H5	−0.1446	0.2194	0.0281	0.032*	
C6	0.14082 (19)	0.22248 (6)	0.12410 (12)	0.0260 (3)	
H6	0.1484	0.2691	0.1298	0.031*	
C7	0.48613 (18)	0.21894 (6)	0.23753 (11)	0.0245 (2)	
H7	0.4946	0.2655	0.2313	0.029*	
C8	0.8228 (2)	0.29167 (6)	0.32415 (13)	0.0273 (3)	
H8A	0.7962	0.2998	0.2284	0.041*	
H8B	0.9595	0.3055	0.3571	0.041*	
H8C	0.7279	0.3168	0.3695	0.041*	
C9	0.94915 (18)	0.18717 (6)	0.42531 (11)	0.0236 (2)	
C10	0.91435 (19)	0.11392 (6)	0.44594 (13)	0.0266 (3)	
H10A	0.7816	0.1076	0.4756	0.032*	
H10B	0.9145	0.0905	0.3605	0.032*	
C11	1.0726 (2)	0.08513 (6)	0.54718 (13)	0.0259 (3)	
C12	1.03317 (13)	0.06569 (4)	0.68966 (8)	0.0410 (3)	0.671 (2)
H12	0.9132	0.0677	0.7291	0.049*	0.671 (2)
S1A	1.03317 (13)	0.06569 (4)	0.68966 (8)	0.0410 (3)	0.329 (2)
C13	1.2424 (3)	0.04236 (7)	0.74842 (14)	0.0396 (3)	
H13	1.2706	0.0278	0.8378	0.048*	
C14	1.3836 (2)	0.04368 (7)	0.66669 (16)	0.0395 (3)	
H14	1.5174	0.0298	0.6934	0.047*	
N1	0.63255 (15)	0.18647 (5)	0.29910 (10)	0.0232 (2)	
N2	0.80022 (16)	0.22142 (5)	0.35017 (10)	0.0236 (2)	
O1	1.10231 (14)	0.21470 (5)	0.47402 (9)	0.0312 (2)	
Cl1	−0.26110 (5)	0.08829 (2)	−0.01956 (3)	0.03259 (11)	
S1	1.30619 (7)	0.07115 (3)	0.51710 (5)	0.03532 (17)	0.671 (2)
C12A	1.30619 (7)	0.07115 (3)	0.51710 (5)	0.03532 (17)	0.329 (2)
H12A	1.3712	0.0770	0.4405	0.042*	0.329 (2)

### (IV) Atomic displacement parameters (Å^2^)

**Table:**

	*U*^11^	*U*^22^	*U*^33^	*U*^12^	*U*^13^	*U*^23^
C1	0.0233 (6)	0.0282 (6)	0.0185 (5)	0.0020 (5)	0.0043 (4)	0.0013 (4)
C2	0.0234 (6)	0.0285 (6)	0.0252 (6)	0.0048 (5)	0.0019 (4)	0.0040 (5)
C3	0.0269 (6)	0.0266 (6)	0.0293 (6)	0.0026 (5)	0.0024 (5)	0.0024 (5)
C4	0.0214 (6)	0.0320 (6)	0.0220 (5)	0.0012 (5)	0.0031 (4)	0.0006 (5)
C5	0.0235 (6)	0.0321 (6)	0.0242 (6)	0.0077 (5)	0.0034 (5)	0.0020 (5)
C6	0.0273 (6)	0.0259 (6)	0.0253 (6)	0.0047 (5)	0.0054 (5)	0.0012 (4)
C7	0.0268 (6)	0.0260 (6)	0.0212 (5)	0.0016 (5)	0.0052 (4)	0.0000 (4)
C8	0.0329 (7)	0.0230 (6)	0.0257 (6)	−0.0020 (5)	0.0027 (5)	0.0011 (4)
C9	0.0260 (6)	0.0250 (6)	0.0196 (5)	−0.0025 (5)	0.0025 (4)	−0.0024 (4)
C10	0.0261 (6)	0.0239 (6)	0.0283 (6)	−0.0027 (5)	−0.0026 (5)	−0.0009 (5)
C11	0.0279 (6)	0.0225 (6)	0.0261 (6)	−0.0031 (5)	−0.0008 (5)	−0.0014 (4)
C12	0.0490 (5)	0.0350 (4)	0.0364 (4)	−0.0036 (3)	−0.0046 (3)	0.0007 (3)
S1A	0.0490 (5)	0.0350 (4)	0.0364 (4)	−0.0036 (3)	−0.0046 (3)	0.0007 (3)
C13	0.0586 (10)	0.0293 (7)	0.0281 (6)	−0.0053 (6)	−0.0050 (6)	0.0039 (5)
C14	0.0347 (7)	0.0341 (7)	0.0459 (8)	0.0038 (6)	−0.0088 (6)	−0.0062 (6)
N1	0.0231 (5)	0.0272 (5)	0.0191 (5)	−0.0023 (4)	0.0026 (4)	−0.0017 (4)
N2	0.0256 (5)	0.0227 (5)	0.0221 (5)	−0.0025 (4)	0.0019 (4)	−0.0009 (4)
O1	0.0304 (5)	0.0282 (5)	0.0325 (5)	−0.0069 (4)	−0.0050 (4)	−0.0004 (4)
Cl1	0.02389 (17)	0.03644 (19)	0.03586 (19)	0.00052 (12)	−0.00205 (12)	−0.00053 (12)
S1	0.0304 (3)	0.0429 (3)	0.0313 (3)	0.00544 (19)	−0.00098 (18)	0.00246 (18)
C12A	0.0304 (3)	0.0429 (3)	0.0313 (3)	0.00544 (19)	−0.00098 (18)	0.00246 (18)

### (IV) Geometric parameters (Å, º)

**Table:**

C1—C6	1.3961 (17)	C9—N2	1.3693 (16)
C1—C2	1.3987 (18)	C9—C10	1.5241 (17)
C1—C7	1.4656 (17)	C10—C11	1.5007 (17)
C2—C3	1.3851 (18)	C10—H10A	0.9900
C2—H2	0.9500	C10—H10B	0.9900
C3—C4	1.3922 (18)	C11—S1A	1.5564 (15)
C3—H3	0.9500	C11—C12	1.5564 (15)
C4—C5	1.3834 (19)	C11—C12A	1.6679 (14)
C4—Cl1	1.7484 (13)	C11—S1	1.6679 (14)
C5—C6	1.3852 (18)	C12—C13	1.5337 (19)
C5—H5	0.9500	C12—H12	0.9500
C6—H6	0.9500	S1A—C13	1.5337 (19)
C7—N1	1.2805 (16)	C13—C14	1.343 (2)
C7—H7	0.9500	C13—H13	0.9500
C8—N2	1.4619 (16)	C14—C12A	1.6388 (16)
C8—H8A	0.9800	C14—S1	1.6388 (16)
C8—H8B	0.9800	C14—H14	0.9500
C8—H8C	0.9800	N1—N2	1.3778 (14)
C9—O1	1.2210 (15)	C12A—H12A	0.9500
			
C6—C1—C2	118.70 (11)	C11—C10—H10A	109.3
C6—C1—C7	118.62 (11)	C9—C10—H10A	109.3
C2—C1—C7	122.67 (11)	C11—C10—H10B	109.3
C3—C2—C1	120.66 (11)	C9—C10—H10B	109.3
C3—C2—H2	119.7	H10A—C10—H10B	108.0
C1—C2—H2	119.7	C10—C11—S1A	122.91 (11)
C2—C3—C4	118.94 (12)	C10—C11—C12	122.91 (11)
C2—C3—H3	120.5	C10—C11—C12A	123.03 (10)
C4—C3—H3	120.5	S1A—C11—C12A	114.06 (9)
C5—C4—C3	121.82 (12)	C10—C11—S1	123.03 (10)
C5—C4—Cl1	118.56 (10)	C12—C11—S1	114.06 (9)
C3—C4—Cl1	119.61 (10)	C13—C12—C11	100.36 (9)
C4—C5—C6	118.35 (11)	C13—C12—H12	129.8
C4—C5—H5	120.8	C11—C12—H12	129.8
C6—C5—H5	120.8	C13—S1A—C11	100.36 (9)
C5—C6—C1	121.52 (12)	C14—C13—C12	116.37 (12)
C5—C6—H6	119.2	C14—C13—S1A	116.37 (12)
C1—C6—H6	119.2	C14—C13—H13	121.8
N1—C7—C1	120.55 (11)	C12—C13—H13	121.8
N1—C7—H7	119.7	C13—C14—C12A	114.47 (12)
C1—C7—H7	119.7	C13—C14—S1	114.47 (12)
N2—C8—H8A	109.5	C13—C14—H14	122.8
N2—C8—H8B	109.5	S1—C14—H14	122.8
H8A—C8—H8B	109.5	C7—N1—N2	117.49 (11)
N2—C8—H8C	109.5	C9—N2—N1	117.06 (10)
H8A—C8—H8C	109.5	C9—N2—C8	120.64 (10)
H8B—C8—H8C	109.5	N1—N2—C8	122.29 (10)
O1—C9—N2	120.92 (11)	C14—S1—C11	94.72 (8)
O1—C9—C10	121.88 (11)	C14—C12A—C11	94.72 (8)
N2—C9—C10	117.20 (10)	C14—C12A—H12A	132.6
C11—C10—C9	111.45 (10)	C11—C12A—H12A	132.6
			
C6—C1—C2—C3	−0.44 (18)	C10—C11—S1A—C13	−178.00 (11)
C7—C1—C2—C3	−179.43 (11)	C12A—C11—S1A—C13	1.98 (11)
C1—C2—C3—C4	0.27 (19)	C11—C12—C13—C14	−1.60 (14)
C2—C3—C4—C5	0.17 (19)	C11—S1A—C13—C14	−1.60 (14)
C2—C3—C4—Cl1	179.53 (9)	S1A—C13—C14—C12A	0.63 (17)
C3—C4—C5—C6	−0.41 (19)	C12—C13—C14—S1	0.63 (17)
Cl1—C4—C5—C6	−179.78 (9)	C1—C7—N1—N2	177.91 (10)
C4—C5—C6—C1	0.23 (18)	O1—C9—N2—N1	−179.97 (11)
C2—C1—C6—C5	0.18 (18)	C10—C9—N2—N1	−0.43 (15)
C7—C1—C6—C5	179.22 (11)	O1—C9—N2—C8	1.27 (18)
C6—C1—C7—N1	172.64 (11)	C10—C9—N2—C8	−179.20 (11)
C2—C1—C7—N1	−8.36 (17)	C7—N1—N2—C9	174.66 (10)
O1—C9—C10—C11	8.78 (17)	C7—N1—N2—C8	−6.59 (16)
N2—C9—C10—C11	−170.75 (11)	C13—C14—S1—C11	0.60 (13)
C9—C10—C11—S1A	106.71 (12)	C10—C11—S1—C14	178.36 (11)
C9—C10—C11—C12	106.71 (12)	C12—C11—S1—C14	−1.62 (10)
C9—C10—C11—C12A	−73.26 (14)	C13—C14—C12A—C11	0.60 (13)
C9—C10—C11—S1	−73.26 (14)	C10—C11—C12A—C14	178.36 (11)
C10—C11—C12—C13	−178.00 (11)	S1A—C11—C12A—C14	−1.62 (10)
S1—C11—C12—C13	1.98 (11)		

### (IV) Hydrogen-bond geometry (Å, º)

Cg1 is the centroid of the benzene ring.

**Table:**

*D*—H···*A*	*D*—H	H···*A*	*D*···*A*	*D*—H···*A*
C5—H5···O1^i^	0.95	2.18	3.1250 (15)	172
C3—H3···Cl1^ii^	0.95	2.95	3.8044 (14)	151
C12—H12···Cl1^iii^	0.95	2.98	3.7960 (10)	145
C8—H8*C*···*Cg*1^iv^	0.98	2.73	3.5592 (14)	142

Symmetry codes: (i) *x*−3/2, −*y*+1/2, *z*−1/2; (ii) −*x*, −*y*, −*z*; (iii) *x*+1, *y*, *z*+1; (iv) *x*−1/2, −*y*+1/2, *z*+1/2.
